# ORAL HEALTH IN LONG-TERM CARE FACILITIES: A SYSTEMATIC REVIEW

**DOI:** 10.1093/geroni/igae098.2490

**Published:** 2024-12-31

**Authors:** Xiaochuan Wang, Eman Zaman

**Affiliations:** University of Central Florida, Orlando, Florida, United States; University of Central Florida, Orlando, Florida, United States

## Abstract

Oral health in long-term care facilities (LTCFs) is becoming an increasing concern as the aging population grows bigger. Recent research indicates that less than 20% of older adults living in LTCFs receive proper dental care. This gap is expected to continue to expand due to various reasons such as oral health being deemed less important in healthcare culture, especially for LTCF residents who may already have multiple other medical conditions. Inadequate oral care can adversely affect one’s mental, physical, and social well-being, causing disproportionate impacts on the vulnerable LTCF resident population. Thus, it is pivotal to understand the current status of oral health care in LTCFs, and LTCF workforce’s roles, competency, and preparedness in providing such care. To address this gap, a systematic review of existing literature was conducted using PRISMA guidelines. We searched electronic databases (e.g., PubMed, ProQuest) to identify peer-reviewed articles published in English between 2013 and 2023. A total of 1101 unduplicated publications emerged in the search. After screening articles for relevance based on titles and abstracts, 22 were reviewed in full text. Sixteen articles were included in this review. Findings demonstrate that residents in LTCFs suffer from poor oral care as its significance is overlooked. The articles suggest solutions such as integrated care, expanded insurance access, and further research on oral hygiene in LTCFs. Findings underscore the importance of oral health education programs. Proper training and increased awareness is needed to prevent further health issues beyond mouth to improve oral and overall health of LTCF residents.

